# Magnetic Resonance Imaging of Peritoneal Carcinomatosis: Evaluation of High b-Value Computed Diffusion-Weighted Imaging

**DOI:** 10.3390/curroncol29070364

**Published:** 2022-06-29

**Authors:** Maxime Ablefoni, Jakob Leonhardi, Constantin Ehrengut, Matthias Mehdorn, Robert Sucher, Ines Gockel, Timm Denecke, Hans-Jonas Meyer

**Affiliations:** 1 Department of Paediatric Radiology, University of Leipzig, Liebigstraße 20a, 04103 Leipzig, Germany; maxime.ablefoni@medizin.uni-leipzig.de; 2 Department of Diagnostic and Interventional Radiology, University of Leipzig, Liebigstraße 20, 04103 Leipzig, Germany; jakob.leonhardi@medizin.uni-leipzig.de (J.L.); constantin.ehrengut@medizin.uni-leipzig.de (C.E.); timm.denecke@medizin.uni-leipzig.de (T.D.); 3 Department of Visceral, Transplant, Thoracic and Vascular Surgery, University Hospital of Leipzig, 04103 Leipzig, Germany; matthias.mehdorn@medizin.uni-leipzig.de (M.M.); robert.sucher@medizin.uni-leipzig.de (R.S.); ines.gockel@medizin.uni-leipzig.de (I.G.)

**Keywords:** computed diffusion-weighted imaging, high-b-value, peritoneal carcinomatosis

## Abstract

Over the last few years, diffusion-weighted imaging (DWI) has become increasingly relevant in the diagnostic assessment of peritoneal carcinomatosis. The aim of this study was to investigate the benefits of high-b DWI (c-DWI) compared to standard DWI in patients with peritoneal carcinomatosis. A cohort of 40 patients with peritoneal carcinomatosis were included in this retrospective study. DWI was performed with b-values of 50, 400, and 800 or 1000 s/mm² on a 1.5-T magnetic resonance imaging (MRI) scanner. C-DWI was calculated using a mono-exponential model with high b-values of 1000, 2000, 3000, 4000, and 5000 s/mm². All c-DWI images with high b-values were compared in terms of volume, detectability of peritoneal lesions, and image quality with the DWI sequence acquired with a b-value of 800 or 1000 s/mm² by two readers. In the group with a b-value of 800 s/mm², there was no statistically significant difference in terms of lesion volume. In the second group with a b-value of 1000 s/mm², peritoneal carcinomatosis lesions were statistically significantly larger than in the c-DWI with a- high b-value of 2000 s/mm² (median 7 cm³, range 1–26 cm³vs. median 6 cm³, range 1–83 cm³, *p* < 0.05). In both groups, there was a marked decrease in the detectability of peritoneal lesions starting at b = 2000 s/mm². In addition, image quality decreased noticeably from c-DWI at b = 3000 s/mm². In both groups, all images with high b-values at b = 4000 s/mm² and 5000 s/mm² were not diagnostically valuable due to poor image quality. The c-DWI technique offers good diagnostic performance without additional scanning time. High c-DWI b-values up to b = 1000 s/mm² provide comparable detectability of peritoneal carcinomatosis compared to standard DWI. Higher b-values over 1500 s/mm² result in lower image quality, which might lead to misdiagnosis.

## 1. Introduction

The peritoneum is a common site of carcinomatosis, not only for many intra-abdominal solid tumours, but also for extraperitoneal tumours. For example, 70% of patients with ovarian cancer, nearly 20% with gastric cancer, and almost 15% with colorectal cancer already present with peritoneal carcinomatosis at initial diagnosis [1,2,3]. Peritoneal carcinomatosis is generally associated with a poor prognosis [2,3,4]. 

Due to the diversity of the small size, morphology, and localization of peritoneal lesions, early and appropriate diagnosis of peritoneal carcinomatosis remains a diagnostic challenge, despite significant advances in imaging techniques. Peritoneal carcinomatosis cannot, therefore, be adequately detected with imaging modalities, and subsequent diagnostic laparoscopy is recommended to confirm or exclude peritoneal carcinomatosis. As peritoneal carcinomatosis is considered an M1-state, it changes therapeutic regimes in a palliative direction. In defined entities, extensive resection, known as cytoreductive surgery (CRS) and subsequent hyperthermic intraperitoneal chemoperfusion (HIPEC), remains the only curative therapeutic intention. 

Imaging plays a key role in assessing peritoneal tumours [1]. Computed tomography (CT) is the reference examination for the evaluation of patients with peritoneal metastases. However, there was an increase in the diagnostic value of magnetic resonance imaging (MRI) in the last few years due to its high soft tissue contrast [5,6,7]. Especially for small lesions, MRI shows a high sensitivity of up to 90% [1]. For standard-of-care imaging comprising CT, PET, and MRI, only a sensitivity of 54% was reported in a recent prospective analysis [6]. 

Recent studies investigated the applicability and utility of diffusion-weighted MRI (DWI) in peritoneal carcinomatosis [8,9]. Consistently, DWI was shown to increase accuracy in the detection of peritoneal carcinomatosis. It often shows diffusion restriction and appears as hyperintense lesions on a diffusion-weighted image. As a result, this leads to increased contrast between the hyperintense malignant tissue and the surrounding hypointense normal tissue [3]. However, some tumour entities, such as well-differentiated adenocarcinomas, may have a hypointense signal at high b-values due to lower cell density [10]. 

The very few studies assessing the diagnostic value of DWI analysed high b-value images up to 1000 s/mm² [11]. At the time of writing, data on the diagnostic benefit of high b-values above 1000 s/mm² are still lacking.

Especially in the field of oncology, the diagnostic potential of computed DWI (c-DWI) showed promising results [12,13,14]. C-DWI represents a mathematical post-processing technique that creates virtual high b-value images with the use of at least two distinct lower b-values from real acquired DWI data [15,16]. Increased b-values can achieve higher diffusion effects and a better signal-to-noise ratio (SNR) by using input data with a shorter echo time (TE). Thus, the scan time can be kept short and high b-values can be generated without additional scan time [15]. As such, in the diagnosis of tumours, such as prostate cancer, it was demonstrated that images with higher b-values exhibited higher contrast ratios when compared with images with standard b-values [17]. A further diagnostic capability of c-DWI at higher b-values is the reduced T2 shine-through effect compared with standard DWI, which may help to differentiate between cystic lesions and malignant lesions [15]. To the best of our knowledge, although the diagnostic capabilities of c-DWI are promising, there are no data on the diagnostic value of c-DWI with higher b-values over 1000 s/mm² for the diagnosis of peritoneal carcinomatosis.

Therefore, the aim of this present study was to assess the diagnostic benefit of high b-value c-DWI compared to acquired DWI in patients with peritoneal carcinomatosis and to compare high b-value images in terms of their conspicuity and extent of peritoneal lesions.

## 2. Materials and Methods

### 2.1. Patient Population

All patients with known or suspected peritoneal carcinomatosis were retrospectively screened between 4/2017 and 10/2021 in a tertiary referral centre (University Hospital of Leipzig). Inclusion criteria were (1) a contrast-enhanced MRI, including axial DWI and T2-weighted (w) sequence; (2) histopathological confirmation of the primary tumour; (3) confirmation of peritoneal carcinomatosis in a clinical setting due to follow-up staging investigations or diagnostic laparoscopy/explorative surgery. 

### 2.2. Magnetic Resonance Imaging Studies

In all cases, MRI was performed with a 1.5-T MR scanner (Aera, Siemens Healthcare, Erlangen, Germany) with a standardised protocol including axial DWI, and fat-saturated contrast-enhanced T1w-images. All patients were administered a standard dose of 1 mmol/mL Gadovist (Gadobutrol, Bayer HealthCare Pharmaceuticals, Berlin, Germany) as an intravenous injection at a flow rate of 1–2 mL/s, followed by a 20 mL saline flush.

Axial DWI-images were acquired with either b-values of 50, 400, and 800 s/mm² (*n* = 21, 2.5% of all patients) or 50, 400, and 1000 s/mm² (*n* = 19, 47.5% of all patients). 

The computed higher b-values of 1000, 2000, 3000, 4000, and 5000 s/mm² were generated with the postprocessing software “Philips IntelliSpace Portal” (version 11; Philips, Amsterdam, The Netherlands) using the application “MR Advanced Diffusion Analysis” (Philips Health System, Hamburg, Germany). This tool employs a mono-exponential model to generate images with high b-values. The MRI protocol parameters are described in Table 1.

### 2.3. Image Analysis

T2w-images, axial DWI, and fat-saturated contrast-enhanced T1w-images were used to localise peritoneal carcinomatosis. All suspicious lesions for peritoneal carcinomatosis defined by morphological imaging (T2-haste and contrast-enhanced T1-sequences) were evaluated and included in the analysis. These lesions were, subsequently, further analysed with axial c-DWI images. 

To perform qualitative analysis, two readers (M.A. and J.L. with 5 and 3 years of general radiology experience, respectively) conducted visual assessment independently of each other. Acquired DWI with b-values of 800 or 1000 s/mm² and c-DWI with b-values of 1000, 2000, 3000, 4000, and 5000 s/mm² were analysed, in each case. 

**Figure 1:**
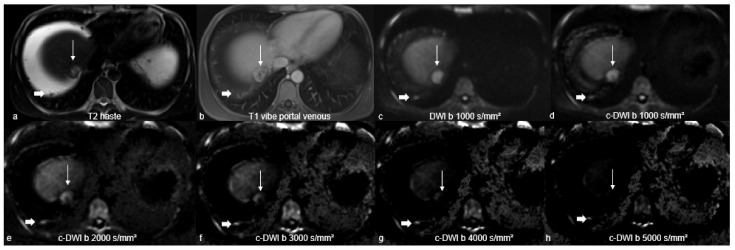
A representative patient of the patient sample with peritoneal carcinomatosis with primary cancer of hepatocellular carcinoma located perihepatically (thin arrow) on T2-weighted sequence (**a**), fat-saturated T1-sequence after contrast agent administration (**b**), axially acquired (**c**), and computed diffusion-weighted imaging at high b-values of 1000–5000 s/mm² (**d**–**h**). On DWI with a b-value of 1000 s/mm² (**c**), peritoneal carcinomatosis is as well visualised as on c-DWI with b-values of 1000 (**d**), and is better visualised than on c-DWI images at higher b-values (2000–5000 s/mm²). Starting from high b-value of 2000 s/mm² (**e**), there is an increasing degradation of image quality. Note the additive discrete peritoneal nodules perihepatic (thick arrow) adherent to the peritoneum with concomitant ascites.

The subjective detectability of peritoneal carcinomatosis at different b-values was categorised as follows: (0) worse delineation, (1) same delineation, and (2) better delineation compared with adjacent surrounding tissue. Image quality was also evaluated in terms of possible artefacts. The readers compared b = 1000, b = 2000, b = 3000, b = 4000, and b = 5000 images simultaneously and side by side. Figure 1 provides an explanatory patient of the patient sample. 

For quantitative analysis, the computed DWI images were compared in terms of lesion volume to the acquired DWI images of b = 800/1000 s/mm². Cases with discrepancies in the measurements were re-examined and discussed by both readers until a consensus was obtained. Quantification of peritoneal carcinomatosis volume was semiautomatically calculated with the tumour-tracking tool in the Philips IntelliSpace Portal. The size was assessed on every slide. The ROI was measured along the boundary of each tumour and its entire volume was generated.

In addition, the presence of splenomegaly (larger than 12 cm in craniocaudal diameter), peritoneal enhancement after contrast administration, and omental cake were evaluated for every patient. The latter refers to the continuous omental mass simulating the top of a cake [1].

### 2.4. Statistical Analysis

Statistical analyses were all conducted using SPSS 27.0 (IBM SPSS Statistics for Windows, Armonk, NY, USA: IBM Corp). Data were tested for normal distribution using the Kolmogorov–-Smirnov test. For descriptive analysis, categorical variables are reported as numbers and percentages. Continuous variables are displayed as mean ± standard deviation (SD), if normally distributed, and as median (interquartile range [IQR]). Descriptive data were assessed using chi-square tests or the Mann–Whitney U test. Lesion size comparison on DWI was performed using the Mann–Whitney U test. Correlation analysis was performed with Spearman’s correlation coefficient. Interrater reliability concerning lesion detectability and image quality was calculated using the intraclass coefficient (ICC) for continuous variables and Cohen’s kappa coefficient for categorical variables as follows: <0.20 = poor agreement; 0.210.40 = fair agreement; 0.41–0.60 = moderate agreement; 0.61–0.80 = good agreement; and 0.81–1.00 = excellent agreement. A *p*-value < 0.05 was used for statistical significance for all cases.

## 3. Results

A patient cohort of 40 consecutive patients (*n* = 21 females, 52.5% of all patients) with peritoneal carcinomatosis was identified. The mean age was 63.2 years, ranging from 55 to 70.5 years (Table 2).

Hepatocellular carcinomas accounted for the majority of primary tumours (*n* = 14; 33.3%), followed by colorectal carcinomas (*n* = 8; 19%), cholangiocarcinomas (*n* = 6; 14.3%), and ovarian cancers (*n* = 6; 14.3%). Other primary tumours were rare.

In 97.5% of the detected peritoneal carcinomatosis, an increase in signal intensity was observed in the high b-values of the DWI (Table 3). Most of these cases showed a strong signal increase (92.5%). Nearly two-thirds of the cases (65%) had a pronounced corresponding decrease in the apparent diffusion coefficient (ADC). The resulting mean ADC value was 0.93 × 10^−3^ mm²/s. There was no statistically significant difference between the most frequent primary tumours. Peritoneal enhancement, omental cake, and large volume ascites occurred in fewer than one-third of patients.

### 3.1. Quantitative Analysis

In the group in which c-DWI-images were generated from DWI-images with b-values of 800 s/mm² or less (*n* = 21; 52.5%), there were no statistically significant differences between the sizes of peritoneal carcinomatosis on DWI at b-values of 800 s/mm² and on c-DWI at higher b-values (Table 4; DWI vs. c-DWI b 1000 s/mm² *p* = 0.766; DWI vs. c-DWI b 3000 s/mm², *p* = 0.125).

In the second group, in which c-DWI-images were generated from DWI-images with b-values of 1000 s/mm² or less (*n* = 19; 47.5%), peritoneal carcinomatosis was significantly larger on acquired DWI-images than on c-DWI-images with high b-values of 2000 s/mm² (Table 4; 7 cm³ [1–26] cm³ vs. 6 cm³ [1–83] cm³, *p*< 0.05). Elsewhere, there was no significant difference in comparison with the other high b-values. The interreader agreement was high, with a resulting ICC ranging from 0.67 to 1.

In a subanalysis only investigating hepatocellular carcinoma, there was no statistically significant difference in terms of lesion volume in either group.

### 3.2. Qualitative Analysis

**Figure 2:**
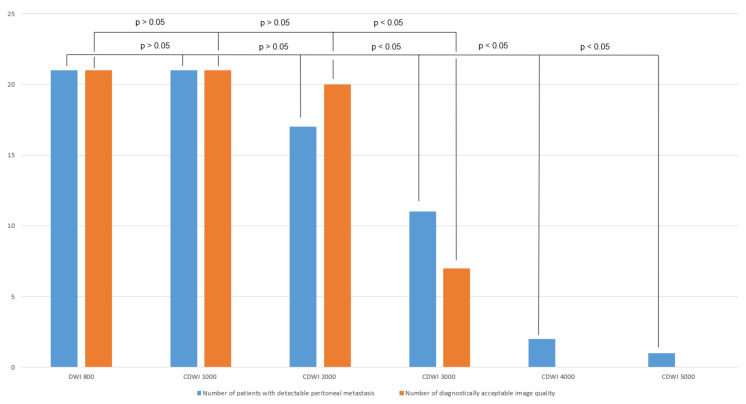
Bar graph showing the number of detected peritoneal carcinomatosis and acceptable image quality in DWI/c-DWI with b-values of 800–5000 s/mm². There were no significant differences between b = 800 and b = 1000–2000 s/mm² in terms of lesion detection or image quality. However, there was a statistically significant decrease beginning at b = 3000 s/mm² in both cases.

Overall, in the b 800-group, all cases of peritoneal carcinomatosis were detected in the acquired DWI and c-DWI at b = 1000 s/mm² (Figure 2; *p* > 0.05). Starting at a b-value of 3000 s/mm², the number of detected peritoneal carcinomatosis on c-DWI decreased statistically significantly (DWI vs. c-DWI b 3000 s/mm²: 21 (100%) vs. 11 (52%), *p* = 0.002).

Image quality decreased significantly in c-DWI images at b = 3000 s/mm² (number of images with diagnostically acceptable quality: DWI b 800 vs. b 3000 s/mm²: 21 (100%) vs. 7 (33%), *p* < 0.01). At b-values of 4000 s/mm² and 5000 s/mm², none of the images were diagnostically evaluable due to poor image quality (Figure 2). In 95% of all patients, the detectability of peritoneal carcinomatosis was the same for DWI-images and c-DWI-images with a high b-value of 1000 s/mm². From the high b-value of 2000 s/mm², there was a marked decrease in detectability, where peritoneal lesions were less visible in 67% of all patients compared with DWI-images with b = 800 s/mm². Interrater reliability ranged from 0.79 to 1.

**Figure 3:**
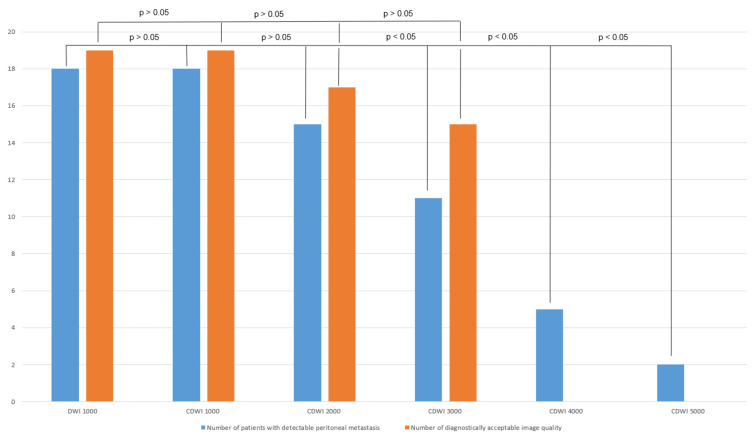
Bar graph showing the number of detected peritoneal carcinomatosis and acceptable image quality in DWI/c-DWI with b-values of 1000–5000 s/mm². There were no significant differences between b = 1000 and b = 1000–2000 s/mm² in terms of detected lesions. There was a statistically significant decrease starting at b = 3000 s/mm². Regarding image quality, no statistically significant differences were found between b = 1000 and b = 1000–3000 s/mm².

Finally, in the b 1000-group, 95% of all peritoneal carcinomatosis were detected in the acquired DWI and c-DWI at b = 1000 s/mm² (Figure 3, *p* > 0.05). At b = 3000 s/mm² and above, the number of peritoneal lesions detected on c-DWI decreased statistically significantly (DWI vs. c-DWI b 3000 s/mm²: 18 (95%) vs. 11 (58%), *p* = 0.016). 

Regarding image quality, there was no statistically significant difference between standard DWI and c-DWI images with b-values of 1000–3000 s/mm². At b-values of 4000 s/mm² and 5000 s/mm², all images were diagnostically unacceptable due to poor image quality. In 79% of all patients, the detectability of peritoneal carcinomatosis was equally good between DWI-images and c-DWI images with a high b-value of 1000 s/mm². From the high b-value of 2000 s/mm², detectability decreased significantly, and peritoneal lesions were less visible in 74% of all patients compared with DWI-images with b = 1000 s/mm². Interrater reliability ranged from 0.79 to 1.

## 4. Discussion

The present study investigated the possible diagnostic benefit of high b-values c-DWI in the diagnosis of peritoneal carcinomatosis. As shown, high b-values up to 1000 s/mm^2^ can potentially be used in clinical routines. As a second key finding, c-DWI images above 2000 s/mm^2^ cannot be recommended due to poor image quality.

Correct diagnosis of peritoneal carcinomatosis is of utmost importance with regard to treatment planning in oncologic patients, because even the diagnostic suspicion of a peritoneal nodule can change a patient’s course from curative intended treatment to a palliative setting [1,2,3,4]. 

The current clinical standard of diagnosis of peritoneal carcinomatosis comprised contrast-enhanced CT, FDG-PET, and MRI. In a very interesting study, the diagnostic abilities of PET-MRI were evaluated [6]. The PET-MRI showed a higher sensitivity compared with clinical standard imaging (0.97; 95% CI 0.86–1.00) compared with 0.54; 95% CI 0.37–0.71, *p* < 0.001, without a difference in specificity. 

There is no doubt regarding the clinical benefit of DWI in abdominal oncological imaging [8]. Yet, there are still uncertainties regarding the definition of the best pair of b-values, which can change the diagnostic abilities of DWI. 

There is a growing interest in c-DWI around oncologic imaging as it can provide high b-value images with novel aspects regarding tissue contrast [15,17]. The principal hypothesis is that the generated high b-value images allow a better lesion contrast with a reduced T2 shine-through effect compared with standard DWI [15,17]. Due to the higher cellularity of malignant tumours, the diffusion restriction can be better visualised by high b-value images, as high b-value images are more sensitive to kurtosis effects [15,17]. 

For ischemic stroke imaging, reliable data were published indicating that high b-value DWI can better display diffusion restriction, which was shown for acquired and computed images alike. Notably, the b-value of 2000 s/mm^2^ had the best image quality in a recent analysis [16].

Early on, it was reported that MRI with inclusion of DWI has a higher sensitivity than without (0.88 versus 0.93) for diagnosis of peritoneal carcinomatosis [9]. Moreover, it was reported that DWI with different b-values of 400 versus 800 s/mm² can detect different amounts of peritoneal tumours [18].

Notably, the addition of DWI into the MRI protocol allows for a better interreader agreement and seems to result in a higher accuracy for unexperienced readers [11]. This effect might be even higher for the c-DWI sequence with a b-value of 1000 s/mm². 

In a recent meta-analysis, 10 studies with a total of 353 patients were included to elucidate the diagnostic accuracy of DWI for peritoneal carcinomatosis [19]. The pooled sensitivity of DWI for peritoneal tumours was 89% (95%CI: 83–93%), and the pooled specificity was 86% (95% CI: 79–91%) [19]. Notably, the study pooled results for all available primary tumours. However, none of the studies used higher b-values above 1000 s/mm² and the meta-analysis did not adjust for different b-values. 

The present results are the first to employ higher b-value DWI in the diagnosis of peritoneal carcinomatosis. For other tumour entities, very promising results were reported for diagnostic improvements owing to this new technique. As such, for prostate cancer, a high b-value DWI of 1500 s/mm^2^ up to 2000 s/mm^2^ is recommended for the standard MRI protocol due to its superior diagnostic abilities compared with standard DWI [20]. Moreover, there are even reports that the c-DWI 2000 s/mm^2^ image might be superior with regard to image quality compared with an actual acquired high b-value image [21]. Similar results were reported for breast cancer patients with c-DWI-images up to 2000 s/mm² in one study and 2500 s/mm² in another [22,23].

For pancreatic cancer, it was only recently published that c-DWI images with b-values of 1500 and 2000 s/mm^2^ are superior in visualisation compared with standard DWI [24]. Especially for these patients, better visualisation, not only of the primary tumour as analysed in the mentioned study, but also the diagnosis of peritoneal carcinomatosis, can be of the utmost importance. Further analyses are needed, especially for this tumour type, using c-DWI.

Only two reports were published regarding c-DWI in liver imaging [12,25]. Kawahara et al. evaluated the diagnostic benefit of c-DWI b-value images of 1000 s/mm^2^, based on 56 patients with hepatic metastases [25], and in another study, the b-values up to 5000 s/mm² were evaluated [12]. Both studies showed that combined c-DWI of 1000 s/mm^2^ is superior to acquired lower b-value DWI alone. 

Compared with other studies, the number of genitourinary cancers as primary tumours is relatively low [9,26]. It should be taken into consideration that the present results are based more upon patients with gastrointestinal and hepatobiliary-caused peritoneal carcinomatosis.

There are some limitations of the present study to address. First, it is a retrospective analysis with possible inherent bias. Furthermore, the patient cohort is relatively small. However, similar studies investigating the diagnostic abilities of DWI had comparable sample sizes [19]. Second, the reading was performed with the knowledge that peritoneal carcinomatosis was present, which could have an influence on the results. Third, due to the study design, we could not compare the c-DWI images to actually acquired high b-value images. It is, therefore, not known whether acquired high b-value images are superior compared to standard DWI and c-DWI images. Fourth, we could not perform subanalyses for primary tumours other than hepatocellular carcinoma due to the small sample size.

## 5. Conclusions

C-DWI images with high b-values up to b = 1000 s/mm² show similar detectability of peritoneal carcinomatosis compared to standard DWI. Higher b-values lead to an increasing deterioration of image quality, which may lead to misdiagnosis. Thus, the c-DWI technique could significantly reduce scanning time by using DWI-images with lower b-values as input data for the production of c-DWI images with high b-values up to b = 1000 s/mm².

## Figures and Tables

**Table 1:** Sequence parameters of axial diffusion-weighted imaging (DWI), T2-weighted and fat-saturated T1-weighted images.

1.5 T MRI Scanner			
Parameters	DWI	T2-Haste	T1-Fat-Saturated
FOV [mm × mm]	295 × 449	312 × 400	300 × 400
Matrix	134 × 88	320 × 200	320 × 180
ST [mm]	5	5	3
number of slices	114	40	72
TR [ms]	7750	1100	3.56
TE [ms]	50.5	119	1.36
Flip angle [°]	90	160	10
b-values [s/mm²]	(*n* = 21; 52.5%) 50, 400, and 800		
	(*n* = 19; 47.5%) 50, 400, and 1000		

Abbreviations: FOV = field of view; ST = slice thickness; TR = repetition time; TE = echo time.

**Table 2:** Overview of the patient sample.

Characteristics	All Patients (*n* = 40)	
	*n*	%
female	21	52.5
male	19	47.5
age (years)	mean 63.2	range 55–70.5
primary tumor		
hepatocellular carcinoma	14	33.3
colorectal cancer	8	19
cholangiocarcinoma	6	14.3
ovarian cancer	6	14.3
pancreatic cancer	2	4.8
gastric cancer	1	2.4
breast cancer	1	2.4
appendiceal adenocarcinoma	1	2.4

**Table 3:** MRI-features and associated findings in peritoneal carcinomatosis.

Peritoneal Carcinomatosis		
**Imaging characteristics**	***n***	**%**
	40	100
**DWI characteristics**		
hyperintensity on high b-value DWI	39	97.5
high	37	92.5
intermediate	2	5
none	1	2.5
**ADC characteristics**		
decreased ADC	36	90
low	26	65
intermediate	10	25
none	4	10
ADC value [×10^−3^ mm²/s]	mean 0.93	range 0.69–1.14
**Imaging findings**		
peritoneal enhancement	8	20
presence of discrete nodules	32	80
omental cake	7	17.5
omental cake [mm]	mean 23.5	range 17.3–30.3
ascites	12	27
large volume	10	25
low volume	2	5
no ascites	28	70
splenomegaly	16	40

Abbreviations: ADC = Apparent diffusion coefficient.

**Table 4:** Comparison of lesion volumes between acquired DWI-images and calculated DWI-images.

Imaging Characteristics	DWI	c-DWI b 1000	c-DWI b 2000	c-DWI b3000	c-DWI b 4000	c-DWI b 5000
c-DWI derived from DWI b 800-images						
Volume cm³ [IQR]	1 [1–7.5]	1 [1–6.5]	1 [0–6]	1 [0–6]	not measurable	not measurable
*p*-value (comparison with DWI b 800-images)		0.766	0.062	0.125		
c-DWI derived from DWI b 1000-images						
volume cm³ [IQR]	7 [1–26]	6 [1–26]	6 [1–83]	7 [1–70]	not measurable	not measurable
*p*-value (comparison with DWI b 1000-images)		0.102	0.021	0.051		

## Data Availability

Qualified researchers may request access to patient level data and related study documents, including the clinical study report, study protocol with any amendments, blank case report form, statistical analysis plan, and dataset specifications. Patient level data will be anonymised and study documents will be redacted to protect the privacy of trial participants.
